# Readiness to treat and factors associated with survival of newborns with breathing difficulties in Ethiopia

**DOI:** 10.1186/s12913-019-4390-9

**Published:** 2019-08-07

**Authors:** Wasihun Andualem Gobezie, Patricia Bailey, Emily Keyes, Ana Lorena Ruano, Habtamu Teklie

**Affiliations:** 1 Averting Maternal Death & Disability (AMDD), Columbia University, New York, NY USA; 2 AMDD, Columbia University, New York, NY USA; 3 Research Associate at Global Health Programs, FHI 360, 359 Blackwell Street, Durham, NC 27701 USA; 4 Department of Global Public Health and Primary Care, University of Bergen, Bergen, Norway; 5 Health System and Reproductive Health Research Directorate, Ethiopian Public Health Institute (EPHI), Addis Ababa, Ethiopia

**Keywords:** Newborn asphyxia, Survival of newborns, Infants with breathing difficulties, Readiness to provide newborn resuscitation, Ethiopia

## Abstract

**Background:**

Ethiopia is one of five countries that account for half of the world’s 2.6 million newborn deaths. A quarter of neonatal deaths in Ethiopia are caused by birth asphyxia. Understanding different dimensions of the quality of care for newborns with breathing difficulties can lead to improving service provision environments and practice. We describe facility readiness to treat newborns with breathing difficulties, the extent to which newborn resuscitation is provided, and by modeling the survival of newborns with difficulties breathing, we identify key factors that suggest how mortality from asphyxia can be reduced.

**Methods:**

We carried out a secondary analysis of the 2016 Ethiopia Emergency Obstetric and Newborn Care Assessment that included 3804 facilities providing childbirth services and 2433 chart reviews of babies born with difficulties breathing. We used descriptive statistics to assess health facilities’ readiness to treat these newborns and a binary logistic regression to identify factors associated with survival.

**Results:**

Over one-quarter of facilities did not have small-sized masks (size 0 or 1) to complete the resuscitation kits. Among the 2190 cases with known survival status, 49% died before discharge, and among 1035 cases with better data quality, 29% died. The odds of surviving birth asphyxia after resuscitation increased eightfold compared to newborns not resuscitated. Other predictors for survival were the availability of a newborn corner, born at term or post-term, normal birth weight (≥2500 g) and delivered by cesarean or assisted vaginal delivery.

**Conclusion:**

The survival status of newborns with birth asphyxia was low, particularly in the primary care facilities that lacked the required resuscitation pack. Newborns born in a facility with better data quality were more likely to survive than those born in facilities with poor data quality. Equipping health centers/clinics with resuscitation packs and reducing the incidence of preterm and low birth weight babies should improve survival rates.

## Background

Globally, intrapartum complications that manifest themselves as difficulties to initiate or sustain breathing are one of the major causes of perinatal and neonatal mortality and morbidity in low and middle-income countries (LMICs) [1–5]. Often referred to as birth asphyxia, they account for about a quarter of all neonatal deaths [6]. According to the World Health Organization (WHO), the condition is characterized by an impairment in the exchange of respiratory gases (oxygen and carbon dioxide), which results in hypoxemia and hypercapnia, accompanied by metabolic acidosis [2, 3]. About 3% of live births, or 3.6 million infants, suffer from moderate to severe asphyxia in LMICs. Of those, 23%, or 840,000 newborns die each year [1, 2, 7]. In countries with high neonatal mortality rates, this rate can be eight times higher than in those with low neonatal mortality rates [8, 9].

Neonatal mortality remains a pervasive issue in most LMICs, with about 38% of all cases coming from countries in sub-Saharan Africa. Ethiopia is among the five countries that accounted for half of the 2.6 million newborn deaths in 2016 [1, 10–13]. The country’s neonatal mortality rate of 29/1000 live births has remained relatively stable despite overall improvements in under-five mortality. Nearly a quarter of these neonatal deaths were caused by birth asphyxia [11–13].

With all the challenges mentioned above, simple investments of procuring and distributing neonatal resuscitation equipment (neonatal size self-inflating bag and mask and suction aspirators) to mid-level health facilities like health centers and clinics can avert many infant deaths due to breathing difficulties at birth. In addition, the availability of health workers who attend delivery and trained in Helping Babies Breathe (HBB) is a crucial step to improve survival of these babies from breathing difficulties [14, 15].

Neonatal resuscitation with bag and mask is a highly cost-effective method of addressing the risks of neonatal mortality and severe morbidities from breathing difficulties at birth [16–18]. In resource-limited countries, very simple interventions like drying, stimulating and keeping the babies warm may even be enough to save the lives of babies with breathing difficulties [7, 15, 19, 20].

Currently, there is a need for standardizing evaluations of clinical practices and services for newborns, particularly for preterm and resuscitated babies in newborn intensive care units (NICUs) and delivery rooms [2, 7, 8]. In Ethiopia there are few large-scale analyses on the readiness to provide care to newborns with breathing difficulties at birth or on their survival [7]; those that exist tend to be geographic- or facility-specific studies. Drawing on the WHO quality of care conceptual framework, which addresses eight process-related domains of quality of care, this study presents insights into four of those eight domains. They include “the evidence-based practice of routine care and management of complications,” in our case the management of intrapartum complications with newborn resuscitation, “actionable information systems,” the availability of “competent, motivated human resources” and “essential physical resources” [18]. Our study describes facility readiness to treat newborns born not breathing or with breathing difficulties and the extent to which newborn resuscitation is provided at both hospital level and health centers and clinics. Secondly, by modeling the survival of newborns with difficulties breathing, we identify key factors that suggest how mortality from asphyxia can be reduced.

## Methods

### Study design

This paper is based on a secondary data analysis using the Ethiopia Emergency Obstetric and Newborn Care (EmONC) 2016 Assessment, which was a cross-sectional census of facilities that provided childbirth services in the 12 months prior to the assessment [21]. All 9 regions and 2 city administrations of Ethiopia were included as were all health facilities (public, private for-profit or private not-for-profit) and in urban or rural settings. Referral, general and primary hospitals and maternal and child health (MCH) specialty centers made up one group of health facilities (hospitals) while health centers, MCH specialty clinics and higher clinics (health centers/clinics) made up a second group. All were targeted as appropriate birthing sites according to government authorities.

For the case reviews, data collectors systematically identified the two most recent cases of neonates with breathing difficulties that occurred at each facility in the last 12 months. Not all facilities provided such cases as some facilities reported none had occurred or they were unable to provide documentation of cases in the 12 months prior to the survey. One year was arbitrary but the same time period was used for all maternal and newborn case reviews. The total convenience sample of the reviewed cases was 2433. For some analyses, we used only 1035 cases that had complete information on newborn outcomes and predictors of the newborn outcome. Data collection occurred from May to November 2016.

### Tools/primary variables

The service readiness data for this paper came from different thematically based modules within the assessment such as human resources and equipment, supplies and drugs. The mortality analysis was based on the case-review module that was tested in the 2016 assessment.

#### Readiness

We measured facility readiness to provide newborn resuscitation by determining the availability of at least one health worker known to provide newborn resuscitation and the availability of resuscitation equipment (mucus extractor or suction aspirator), neonatal face masks – size 0 or 1, and neonatal size self-inflating bag. Actual provision of resuscitation with a bag and mask was defined as having been performed at least once in the 3-month reference period prior to the assessment.

#### Case reviews of newborns with difficulties breathing

Maternal and newborn characteristics were extracted from patient records and included birth weight, gestational age, duration of labor, mode of delivery, whether the mother had an obstetric complication in the index pregnancy, and the outcome of the newborn (survived/alive at discharge or dead). Treatment variables included type of resuscitation used (if at all) and whether the baby was given oxygen.

### Data analysis

Descriptive statistics like frequencies and cross tabulations were used to show facility readiness to provide resuscitation as well as actual provision. A binary logistic regression model was used to identify factors associated with survival among newborns with breathing difficulties at birth. SPSS version 22 was used for both descriptive and regression analyses.

A total of 2433 newborn cases with breathing difficulties were reviewed. However, 2190 cases had information on newborn outcome while only 1035 cases had complete information on newborn outcome and its predictors. These cases were included in the logistic regression model. The dependent variable was birth outcome (alive at discharge or dead, with dead as the reference category), and the independent variables included facility characteristics (facility type where delivery took place, readiness of a facility to provide newborn resuscitation with bag and mask, and availability of a newborn corner [a small room or corner of the delivery room designed to provide immediate newborn care including resuscitation, warmth and care for sick newborns]. Both maternal and newborn characteristics at admission (gestational age, mode of delivery, birth weight) and treatment (oxygen and resuscitation) were also included as independent variables. Multicollinearity testing demonstrated high tolerance values and low variance inflation factors (all less than 1.5).

As Kleinbaum suggested for cross-sectional data [22], survival among newborns was analyzed first with a bivariate binary logistic regression to determine whether each selected factor was significantly associated with birth outcome, and then a multivariate binary logistic regression model was fitted with backward conditional entry of those factors that were significant in the bivariate analysis. A 95% confidence interval (CI) that did not contain the null value, 1, indicated statistical significance. Goodness of fit for both the bivariate and multivariate models (Chi-squares) were tested and significant at *p* < 0.05.

### Ethical considerations

The 2016 EmONC assessment received ethical clearance from the Ministry of Health and the Institutional Review Board (IRB) of the Ethiopian Public Health Institute (EPHI), who was contracted to conduct the EmONC assessment. Before data collection started, a letter requesting support was submitted to each health facility. Throughout the study period, data confidentiality and security were maintained.

## Results

### Readiness of health facilities to provide newborn resuscitation with bag and mask and recent performance

Almost all health facilities that provided delivery services in 2016 had at least one health worker who could provide newborn resuscitation and immediate newborn care (immediate drying, skin-to-skin contact, delayed cord clamping, exclusive breastfeeding within the hour, etc.) (Table 1). Similarly, the availability of a health worker trained in Basic Emergency Obstetric and Newborn Care (BEmONC) was present in over 80% of both hospitals and health centers/clinics. Training of health workers in Essential Newborn Care (ENC) and Helping Babies Breathe (HBB) was also reported by close to two-thirds of the health facilities.

**Table 1:** Percent of facilities ready to provide and perform newborn resuscitation, by readiness characteristics and facility type

Readiness to provide newborn resuscitation with bag and mask	Overall			Facilities where case reviews were taken^a^		
	Total	Hospitals	Health centers	Total	Hospitals	Health centers
	*n* = 3804	*n* = 316	*n* = 3488	*n* = 684	*n* = 154	*n* = 530
	%	%	%	%	%	%
Facility has at least one health worker who:						
Provides newborn resuscitation with bag and mask	98%	99%	98%	99%	99%	99%
Provides immediate newborn care	99%	99%	100%	100%	99%	100%
Is trained in BEmONC	83%	80%	83%	90%	87%	91%
Is trained in ENC/HBB	62%	66%	61%	71%	73%	70%
Facility has equipment						
Mucus extractor/simple suction	89%	96%	88%	95%	97%	95%
Neonatal face masks, sizes 0 or 1	74%	86%	73%	84%	91%	82%
Neonatal self-inflating bag	94%	98%	94%	97%	99%	96%
Suction aspirator (operated by foot or electrically)	21%	80%	16%	34%	84%	19%
Suction catheter (10, 12Ch)	32%	76%	28%	42%	81%	30%
Infant laryngoscope with spare bulb and batteries	7%	48%	3%	15%	53%	4%
Endotracheal tubes (3.5, 3.0, 2.5 mm)	7%	53%	3%	16%	62%	3%
Guideline or protocol for neonatal resuscitation	65%	74%	64%	78%	84%	76%
Oxygen source	15%	90%	9%	30%	91%	12%
Provided newborn resuscitation in the 3 months prior to the assessment	73%	90%	71%	83%	95%	79%
Facility fulfilled summary measure for readiness to provide newborn resuscitation with bag and mask^b^	69%	86%	68%	81%	90%	79%

*Abbreviations: BEmONC* basic emergency obstetric and newborn care, *ENC* essential newborn care, *HBB* Helping Babies Breathe

^a^ Health facilities where 1035 case reviews with complete information were drawn

^b^Summary measure for facility readiness was defined by at least one health worker able to provide newborn resuscitation and presence of equipment (neonatal self-inflating bag and mask size 0 or 1 and suction apparatus (mucus extractor or suction aspirator)

Neonatal size self-inflating bags and mucus extractors were available in over 89% of all facilities, but only 74% had neonatal size masks (size 0 or 1). However, at the national level we found substantial gaps in the availability of manual and electric suction aspirators (21%) and oxygen (15%). Shortage of critical equipment as well as guidelines was reported more frequently in health centers/clinics than in hospitals. To provide context for the case reviews, we selected the 684 facilities from which they came and compared their readiness characteristics with those of all facilities. Consistently, the equipment needed for neonatal resuscitation was more available in the facilities where the cases were drawn than all facilities.

Almost three quarters (73%) of facilities reported that they had actually performed newborn resuscitation in the 3 months prior to the assessment. Performance was lower among health centers/clinics than hospitals. The lack of equipment (small masks, suction aspirators) suggested a greater problem than the shortage of human resources. Performance of the newborn resuscitation was higher in the facilities where the cases were reviewed (83%) than in all facilities.

### Characteristics of newborns with breathing difficulties

As context, the EmONC assessment documented 1,924,330 institutional deliveries, 28,670 of which were stillbirths and 3592 early neonatal deaths (live births who died within 24 h or prior to discharge, whichever came first); these occurred during the calendar year 2015.

Of the 2433 case reviews of newborns with breathing difficulties, 2190 (90%) had information on newborn outcome. Among these, 61% had normal birth weight (≥ 2500 g) (Table 2) but 22% had no information on birth weight. The remaining cases had low and very low birth weight. In addition, women’s gestational age was of interest because low gestational age is a well-known risk factor for birth outcomes and long term development of the child [23, 24]; 45% of cases were at term and only 10% were preterm. Close to half of the cases had no gestational age recorded.

**Table 2:** Percent distribution of newborns with breathing difficulties, by maternal and newborn characteristics and facility type

	All cases with known survival status	Facility type	
		Hospitals/MCH specialty centers	Health centers/clinics
	*n* = 2190	*n* = 351	*n* = 1839
Birth Weight			
Very low birth weight (< 1500)	3%	5%	3%
Low birth weight (1500–2499 g)	14%	16%	14%
Normal birth weight (≥2500 g)	61%	70%	59%
No information	22%	9%	24%
Gestational age			
Preterm (< 37 weeks)	10%	18%	8%
Term (37–42 weeks)	45%	60%	42%
Post-term (> 42 weeks)	0%	1%	0%
No information	45%	21%	49%
Duration of labor			
Precipitate labor (< 1 h)	0%	1%	0%
Normal labor (1–12 h)	23%	25%	22%
Prolonged labor (> 12 h)	3%	10%	2%
No information	74%	64%	76%
Mode of delivery			
Vaginal	90%	68%	94%
Instrumental	3%	9%	2%
Cesarean	3%	18%	0%
No information	4%	5%	4%
Mother experienced obstetric complication (% yes)	6%	18%	4%
Treatment			
Type of resuscitation			
Stimulation	1%	1%	1%
Bag and mask	28%	24%	28%
Both simulation & bag and mask	13%	24%	11%
Intubation	1%	6%	0%
No information /no resuscitation	58%	44%	60%
Oxygen given as needed (% yes)	19%	52%	13%
Newborn outcome			
Alive at discharge	51%	69%	48%
Dead	49%	31%	52%

The duration of labor should be recorded in all patient cards, but in the records reviewed for this study (Table 2), 74% of the cases had no information on labor duration, only 3% were recorded as prolonged (> 12 h) and 23% experienced normal duration of labor, suggesting that the proportion of cases with prolonged/obstructed labor was underestimated.

Spontaneous vaginal delivery was the most common mode of delivery for newborns with breathing difficulties (90%), and was higher among cases from health centers/clinics (94%) than hospitals (68%). Among the hospital case reviews, the cesarean delivery rate was 18%.

Women’s experiences of obstetric complications were also recorded in the patient cards; only 6% of all reviewed cases had evidence of an obstetric complication. This number was 18 and 4% at hospital and health center/clinic levels, respectively.

A large proportion (58%) of the cases had no information regarding what type of resuscitation was performed, if any. Overall, 28% of the cases received resuscitation using a bag and a mask, while health workers used both bag and mask and manual stimulation for another 13%, for a total of 41%. The use of bags and masks was higher in hospitals than in health centers/clinics. Manual stimulation alone was recorded for only 1% of cases in both hospitals and health centers/clinics.

Intubation was performed exclusively in hospitals, where 6% of cases received this more invasive procedure. Endotracheal intubation requires trained staff and availability of suction catheters, laryngoscopes and suction apparatuses. In this study (Table 1), over half (52%) of the hospitals did not have an infant laryngoscope; close to half (47%) lacked endotracheal tubes; and a quarter had no suction catheter. Among all cases of babies with breathing difficulties reviewed and survival status known, oxygen was given to 19%; it was reported more frequently in hospital cases (52%) than in health center/clinic cases (13%). Of the 2190 newborn cases with known outcomes, only 51% were discharged alive. The survival rate of newborns with breathing difficulties was higher in hospitals (69%) than in health centers/clinics (48%) (Table 2).

### Bivariate and multivariate analysis of survival of newborns with breathing difficulties

Preparation for the multivariate analysis required dropping some key variables. As seen in Table 2, nearly ¾ of all cases were missing information on the duration of labor, 58% on type of resuscitation, 45% on gestational age, and 22% on birth weight. Several variables were not associated with survival and thus were not included in the multivariate analysis. These included whether the facility had a newborn intensive care unit and evidence of a maternal obstetric complication.

In the subset of 1035 cases with complete information, approximately 71% were discharged alive from both hospitals and health centers (Table 3). Survival of newborns was higher in facilities that were ready to provide newborn resuscitation (72%) than non-ready facilities (64%). Similarly, survival of babies born in facilities with a newborn corner was higher (77%) than those without a newborn corner (66%). Among term or post-term newborns, 75% survived compared to only half (51%) of preterm infants. Survival was also higher among infants of normal birth weight (> 2500 g) and among those born by cesarean or with instrumental assistance compared to their counterparts (newborns with low birth weight and those born vaginally but unassisted). Close to four-fifths (79%) and 86% of the babies with breathing difficulties who received oxygen or resuscitation with bag and mask, respectively, survived.

**Table 3:** Parameter estimates of the bivariate and multivariate analysis of survival of newborns with breathing difficulties

		Alive at discharge (survived) (*n* = 730)	Dead (*n* = 305)	Unadjusted OR (95% CI)	Adjusted OR (95% CI)
		n(%)	n(%)		
Place of birth	Hospitals (*n* = 253)	177 (70%)	76 (30%)	0.82 (0.71–1.32)	0.68 (0.45–1.06)
	Health centers (*n* = 782) (ref.)	553 (71%)	229 (29%)	1	1
Facility ready to provide newborn resuscitation^a^	Yes (*n* = 851)	612 (72%)	239 (28%)	1.25 (0.90–1.74)	1.08 (0.73–1.60)
	No (*n* = 184) (ref.)	118 (64%)	66 (36%)	1	1
Facility has separate newborn corner	Yes (*n* = 443)	341 (77%)	102 (23%)	**1.75 (1.32–2.31)**	**1.75 (1.23–2.47)**
	No (*n* = 592) (ref.)	389 (66%)	203 (34%)	1	
Gestational age	Term or post-term (*n* = 858)	639 (75%)	219 (25%)	**2.76 (1.98–3.84)**	**1.57 (1.04–2.39)**
	Preterm (*n* = 177) (ref.)	91 (51%)	86 (49%)	1	
Mode of delivery	Cesarean or instrumental (*n* = 95)	78 (82%)	17 (18%)	**2.03 (1.18–3.49)**	**2.09 (1.08–4.03)**
	Vaginal (vertex) (*n* = 940) (ref.)	652 (69%)	288 (31%)	1	1
Birth weight	Normal (> = 2500 g) (*n* = 802)	608 (76%)	194 (24%)	**2.85 (2.11–3.86)**	**2.35 (1.61–3.43)**
	Low birth weight (< 2500 g) (*n* = 233) (ref.)	122 (52%)	111 (48%)	1	1
Oxygen given	Yes (*n* = 285)	226 (79%)	59 (21%)	**1.87 (1.35–2.59)**	0.93 (0.62–1.38)
	No (*n* = 750) (ref.)	504 (67%)	246 (33%)	1	1
Received resuscitation^b^	Yes (*n* = 665)	572 (86%)	93 (14%)	**8.25 (6.11–11.15)**	**8.01 (5.77–11.11)**
	No (*n* = 370), ref.	158 (43%)	212 (57%)	1	1
Newborn outcome		730 (71%)	305 (29%)		

Newborn outcome (Reference category) = dead, *OR* Odds Ratio, *CI* Confidence Interval, CIs highlighted bold are significant at *P* < 0.05

^a^ facility readiness defined by at least one health worker able to provide newborn resuscitation, had resuscitation pack (self-inflating bag and mask size 0 or 1) and suction apparatus (mucus extractor or suction aspirator). ^b^ Includes: stimulation, resuscitation with self-inflating bag and mask and/or intubation

CIs highlighted bold are significant at *p* < 0.05

After controlling for other variables, individual newborn characteristics, facility characteristics and treatment variables were independently associated with newborn survival. Babies who were term or post-term were 57% more likely to survive than preterm infants. Newborns of normal birth weight (≥2500 g) were 2.3 times as likely to survive than those with low birth weight. Facilities with a separate newborn corner were 75% more likely to survive than newborns who were delivered in a facility without a newborn corner. Newborns whose mothers underwent an operative delivery (abdominal or vaginal) were twice as likely to survive than those whose mothers delivered spontaneously. Controlling for other variables, the administration of oxygen did not predict survival, but newborns known to have received any form of resuscitation (stimulation, bag and mask, or intubation) were 8 times as likely to survive than those who received no resuscitation (Table 3).

### Data quality

Complete and updated register books, one of our primary data sources, were not the norm, as we observe in Table 4 below. To examine data quality in the 2016 EmONC assessment we collected information on the existence of registers (for example, labor and delivery and operating theatre registers, where both maternal and newborn outcome data are available).

**Table 4:** Percent of facilities with complete and up-to-date registers (labor and delivery and operating theatre), by facility type

	Labor and delivery register			Operating theatre register		
	Facilities with register	Register complete	Register up-to-date	Facilities with register	Register complete	Register up-to-date
	n	%	%	n	%	%
Facility type						
All facilities	3784	35%	87%	295	63%	81%
Hospitals/MCH specialty centers	315	44%	90%	262	63%	85%
Health centers/ clinics	3469	34%	86%	33	58%	48%

Completeness, defined as routinely filling out all the items that appear as column headings in the labor and delivery register, was only 34, 44%, of health centers/clinics and hospitals, respectively [21]. Hospital operating theatre registers were more complete (63%) than labor and delivery registers. The lack of complete information, not only in case reviews, but also in obtaining service statistics, was universally very low in the country [21].

## Discussion

This secondary data analysis targeted 3804 health facilities, all facilities that had provided childbirth services in 2015, and 2433 case reviews of newborns with breathing difficulties at birth, making it an ideal resource for program planning by the Ministry of Health and its implementing partners.

The most striking result of this study was the low survival rate among newborns born with breathing difficulties or not breathing at all. Although data quality was undermined by incomplete information, 90% of newborns whose cases were reviewed (2190/2433) had information on survival. Among those, 51% were discharged alive, but among a subset of cases for which data quality was more complete, 71% survived (730/1035) The difference between 51 and 71% survival suggests that data quality (completeness of recording) may be a proxy for quality of care. Both statistics are unacceptably high given that most of these deaths could have been prevented. However, the methodology used for case selection made clear that babies that might have been intrapartum stillbirths could be included. Thus, mortality may have been inflated by the inclusion of stillbirths for whom resuscitation would not have been effective.

Despite methodological differences, the mortality of babies with breathing difficulties in this study was high when compared to other studies in Ethiopia and elsewhere (Dire Dawa, Ethiopia – 11% [12]; Jima, Ethiopia – 23% [25]; Nigeria (two studies documenting mortality rates of 18 and 25%) [4, 26]; and South Africa - 20% [20]). However, other studies found even higher mortality (Tigray, Ethiopia – 31% [27]; Nepal (30%) [28]; and 50% in Zambia [29]), though the latter had a small sample size.

Multiple sources over the past decade show that under-five, infant and neonatal mortality indicators have improved [11, 30]. However, the most intransigent portion of neonatal mortality is that which occurs during the first week of life. The assessment showed an 11-fold increase in the number of health facilities with childbirth services since 2008 when a similar assessment was conducted. In addition to the expansion of the health system, facilities in 2016 were better equipped and staffed to intervene on behalf of newborns with breathing difficulties [7, 21]. Nevertheless, gaps still remain that help explain the low survival rate among newborns with breathing difficulties, especially at health centers and clinics.

For facilities to be able to provide high-quality intrapartum care, it is necessary to improve the availability of skilled staff and to ensure consistent stocking of essential drugs, equipment and supplies. This is crucial for the reduction of newborn mortality [31]. The availability of skilled personnel trained in BEmONC, which explicitly includes neonatal resuscitation with bag and mask, was high in both hospitals and health centers/clinics. Almost all facilities had at least one health worker who could provide newborn resuscitation and also immediate newborn care. Although BEmONC training includes neonatal resuscitation, to scale-up service provision this skill area is also provided as a stand-alone training package for nurses and midwives. Despite the high percentage of facilities with health workers with critical newborn care skills, if we look at the percentage of different cadres such as nurses and midwives, only 3 and 57%, respectively, reported training in BEmONC (unpublished data). On top of this, we saw a gap in the availability of health workers trained on essential newborn care and Helping Babies Breathe; over a third of the facilities had no health worker with this training.

Newborn resuscitation with bag and mask was not performed in one-quarter of the facilities assessed, with health centers/clinics being less likely than hospitals to have performed this procedure. Certain types of newborn resuscitation equipment were absent in many of these facilities, namely suction aspirators and facemasks (size 0 or 1). Although, we aggregated the two sizes, they were equally available.

According to the 2012 WHO guidelines on basic newborn resuscitation, stimulation through rubbing the baby’s back 2–3 times is often all that is needed to initiate spontaneous breathing [16]. Among the cases reviewed, hand stimulation was recorded for only 14% of cases,. It is possible that a higher percentage of newborns received hand rubbing but it was not routinely recorded. The odds of surviving birth asphyxia after resuscitation with bag and mask or extra stimulation increased eightfold compared to those not resuscitated at all (*p* < 0.001). This finding was similar to results found in South Africa [20] and Pakistan [32].

Several of the other multivariate findings have also been confirmed by studies elsewhere. Babies born preterm or with low birth weight, often the same infants, are at increased risk of having difficulties breathing at birth. Our study also showed these newborns to be at higher risk of dying. Similar relationships between gestational age and survival have been reported in studies conducted in Ethiopia (Jima) [25], Kenya [15], Ghana [33], Pakistan [34], and Nepal [35]. The same is true of studies looking at the impact of low birth weight [4, 15, 25, 33, 36].

The mode of delivery was also a key variable of interest, and assisted delivery (cesarean or instrumental) doubled the odds of survival for the newborn. It is possible that the babies born by cesarean or by assisted vaginal delivery were born more quickly and were better monitored for fetal distress or duration of labor than babies who delivered vaginally. Other studies have shown mixed results regarding mode of delivery: our results were similar to those from a study in Dire Dawa, Ethiopia) [33], in contrast to studies in South Africa [20], Cameroon [37] and Nigeria [4] that demonstrated higher survival among newborns with a spontaneous vaginal delivery.

### Limitations

Several study design issues presented limitations to this study, especially those related to the case reviews. The case review sample, although systematically selected, was not random and therefore, has undermined the generalizability of these results. The attrition of cases for analysis, due to poor quality of data (incompleteness) further undermined their generalizability. However, we were able to show by comparing the results of all health facilities (*n* = 3804) to those of facilities where case reviews were performed (*n* = 684) and with complete data for the regression analyses that the subset of facilities had more favorable characteristics as far as readiness to provide newborn resuscitation with bag and mask and survival outcomes.

From the bivariate regression analysis, we saw that the proportion of deaths among newborns with breathing problems at birth was similar in hospitals and health centers/clinics (~ 30%). This was in contrast to the larger group of 2190 cases where 52% of health center cases died and only 31% of hospital cases died. This attrition in the cases used for analysis was unfortunate, but informative nonetheless.

Our definition of readiness was designed originally as a minimum set of conditions that had to be met – staffing and equipment – to carry out newborn resuscitation with bag and mask. It is unrealistic to define readiness by the availability of at least one health worker since no one single person can be present 24 h a day 7 days a week. A better solution would be for every person who attends deliveries to have the skills and confidence to safely use a bag and mask. Similarly, we recognize that having either a size 0 mask or a size 1 will fall short if the desired size is not available. We saw great discrepancy in availability of a suction aspirator versus the mucus extractor, with few health centers or clinics having the former. Going forward, readiness criteria could be tailored to different levels of care and with greater scrutiny as to the health worker component.

We also recognize that improvements to the tool are in order. We did not inquire into the precise size of the ventilatory bag. But more importantly, we were unable to separate those cases who were born not breathing from those who were unambiguously born alive but with difficulties breathing. Clinical management is likely to vary depending on this critical observation. Furthermore, we were unable to disaggregate the final outcome (dead or alive) into more nuanced categories that might have helped explain the high mortality. Whether the written records that the data collectors examined during the case review would have reflected these finer points is not clear.

## Conclusion

Ethiopian health facilities appeared to be better staffed to provide routine care and to manage newborns such as breathing problems at birth than they were equipped and supplied to do so. Survival rates were low among babies born not breathing or born with breathing problems, and we saw that low birth weight and preterm births contributed to poor outcomes. In the Ethiopian setting, having a cesarean or an instrumental delivery was protective as was having a newborn corner at the health facility, but intervening with newborn resuscitation was clearly the most important predictor of survival.

A concerted effort to better equip lower- and mid-level facilities with basic equipment and newborn corners is called for so that resuscitation can be provided quickly. All persons involved in delivering a baby should also be competent at using a bag and mask. Although health information systems seem to be in place, for them to be optimally “actionable,” data quality, specifically more complete reporting, is needed, especially at the health center/clinic level. Training and orientation of providers to record and report key information for decision-making and planning are crucial to ending preventable deaths.

## Data Availability

Data for this research paper is available publicly with permission at the ministry of health (MOH) and EPHI offices.

## Abbreviations

BEmONC: Basic emergency obstetric and newborn care
EmONC: Emergency obstetric and newborn care
ENC: Essential newborn care
EPHI: Ethiopian Public Health Institute
HBB: Helping Babies Breathe
IRB: Internal Review Board
LMICs: Low and middle-income countries
MCH: Maternal and child health
WHO: World Health Organization
